# Extended antigen sparing potential of AS03-adjuvanted pandemic H1N1 vaccines in children, and immunological equivalence of two formulations of AS03-adjuvanted H1N1 vaccines: results from two randomised trials

**DOI:** 10.1186/1471-2334-13-435

**Published:** 2013-09-16

**Authors:** Odile Launay, Xavier Duval, Serge Fitoussi, Wolfgang Jilg, Angkool Kerdpanich, May Montellano, Tino F Schwarz, Veerachai Watanveerade, Jürgen J Wenzel, Gerard Zalcman, Vinod Bambure, Ping Li, Adrian Caplanusi, Anuradha Madan, Paul Gillard, David W Vaughn

**Affiliations:** 1 Inserm, CIC BT505; Assistance Publique-Hôpitaux de Paris, Groupe Hospitalier Cochin Broca Hôtel-Dieu, CIC de vaccinologie Cochin Pasteur; Université Paris Descartes, Paris, France; 2 The National clinical vaccine research network (REIVAC), Paris, France; 3 C.I.C Bichat, Paris, France; 4 Mediscis, Poitiers, France; 5 Institute of Medical Microbiology and Hygiene, University of Regensburg, Regensburg, Germany; 6 Infectious Diseases Unit, Department of Pediatrics, Phramongkutklao Hospital, Bangkok, Thailand; 7 Department of Pediatrics, Mary Chiles General Hospital, Manila, Philippines; 8 Central Laboratory and Vaccination Centre, StiftungJuliusspital, Würzburg, Germany; 9 Caen University Hospital – Early Phase Research Center/Centre de Recherche Clinique, Caen, France; 10 GlaxoSmithKline Vaccines, Bangalore, India; 11 GlaxoSmithKline Vaccines, King of Prussia, PA, USA; 12 GlaxoSmithKline Vaccines, Wavre, Belgium; 13 CIC deVaccinologie Cochin Pasteur, Hopital Cochin, 27 Rue du Faubourg St.Jacques, 75679 Paris, France

**Keywords:** H1N1, Pandemic influenza vaccine, Influenza virus, Children, Adults, Persistence, Immunogenicity, Manufacturing capacity, Antigen dose reduction

## Abstract

**Background:**

Pandemic influenza vaccine manufacturing capacity and distribution agility is enhanced through the availability of equivalent antigen-sparing vaccines. We evaluated equivalence in terms of immunogenicity between GlaxoSmithKline Vaccines’ A/California/7/2009 (H1N1)v-like-AS03 vaccines manufactured in Dresden (D-Pan), and Quebec (Q-Pan).

**Methods:**

In two studies, 334 adults 18-60 years of age received 2 doses of D-Pan or Q-Pan containing 3.75 μg haemagglutinin antigen (HA) adjuvanted with AS03_A_ administered 21 days apart, and 209 children 3-9 years of age received 1 reduced dose of D-Panor Q-Pan (0.9 μg HA) or Q-Pan (1.9 μg HA) with AS03_B_. Haemagglutination inhibition (HI) titres were assessed before and 21 days post-vaccination. HI persistence was assessed after 12 months in adults and 6 months in children.

**Results:**

Pre-defined criteria for immunological equivalence of Q-Pan versus D-Pan were achieved in both populations. After one vaccine dose, ≥97.6% of adults and children had HI titres ≥1:40, with increases in titre ≥25.7-fold. CHMP and CBER regulatory acceptance criteria for influenza vaccines were exceeded by all groups in both studies at Day 21. In adults,the percentage with HI titres ≥1:40 at Month 12 was 82.9% (Q-Pan) and 84.0% (D-Pan). In children, the percentages at Month 6 were 75.3.3% (Q-Pan0.9), 85.1% (D-Pan0.9) and 79.3% (Q-Pan1.9). Safety profile of the study vaccines was consistent with previously published data.

**Conclusion:**

Two studies indicate that A/California/7/2009 (H1N1)v-like HA manufactured at two sites and combined with AS03 are equivalent in terms of immunogenicity in adults and children and highly immunogenic. Different HA doses elicited an adequate immune response through 180 days post-vaccination in children 3-9 years of age.

**Trial registration:**

ClinicalTrials.gov: NCT00979407 and NCT01161160.

## Background

In June 2009 the World Health Organisation declared the first influenza pandemic since the 1960s, caused by A/California/7/2009 H1N1 influenza strain [1]. More than 18,000 H1N1-related deaths were reported by July 2010, with infections reported in 214 countries worldwide [2,3]. Vaccination is regarded as the most effective intervention to prevent and attenuate influenza pandemics [4], and by the end of 2009, vaccines targeting the pandemic A/California/7/2009 H1N1 strain had been produced by several manufacturers.

Global influenza antigen manufacturing capacity is limited, and the formulation of H1N1 vaccines with oil-in-water adjuvants using reduced amounts of virus antigen match or surpass immunogenicity compared to unadjuvanted formulations allowing for an increased number of doses from the available antigen bulk (antigen sparing). GlaxoSmithKline Vaccines’ A/California/7/2009 (H1N1)v-like vaccine contains the proprietary Adjuvant System 03 (AS03) which allows a 4-fold reduction in the amount of haemagglutinin antigen (HA) necessary to achieve an adequate immune response in adults [5,6]. The A/H1N1/2009-AS03 influenza vaccine has been demonstrated to be immunogenic, with a clinically acceptable safety profile in adults, adolescents and children [5-10]. A single dose of A/H1N1/2009-AS03 was recommended for adults (3.75 μg HA). A single dose containing half of the adult dose (1.9 μg HA) was recommended for children 6 months to 10 years of age [11].

The A/H1N1/2009-AS03 vaccine was manufactured at two sites: vaccine manufactured in Dresden is licensed as *Pandemrix*™ (D-Pan), and vaccine manufactured in Quebec is licensed as *Arepanrix*™ *H1N1*(Q-Pan), using somewhat different methods of HA preparation. HA for a pre-pandemic H5N1 vaccine manufactured at the Dresden and Quebec sites was shown to be equivalent in terms of immunological outcomes when administered with AS03 to adults [12]. Demonstration of immunological equivalence between D-Pan and Q-Pan H1N1 vaccines would provide reassurance on the comparability of both products, and would allow clinical trial data and post-marketing effectiveness estimates to be extrapolated between each product. We report the results of two clinical studies conducted with D-Pan and Q-Pan H1N1 vaccines that confirmed the equivalence of the two vaccines in terms of immunogenicity in adults and children, and assessed the feasibility of further antigen sparing in children.

## Methods

### Study design

The adult study (113535, NCT00979407) was a Phase III, randomised, controlled study conducted in 7 centres in Germany and France between 12 October 2009 and 4 November 2010. Healthy adults between 18 and 60 years of age were randomised (1:1) to receive 2 doses of either D-Pan or Q-Pan (3.75 μg HA) adjuvanted with AS03_A_ administered 21 days apart (Table 1).

**Table 1:** Study design

**Study**	**Group**	**Vaccine**	**HA dose**	**Schedule**
**Study in adults**	D-Pan	A/California/7/2009 (H1N1)v-like + AS03_A_	3.75 μg	2 doses
18-60 year olds	Q-Pan	A/California/7/2009 (H1N1)v-like + AS03_A_	3.75 μg	2 doses
**Study in children**	D-Pan0.9	A/California/7/2009 (H1N1)v-like + AS03_B_	0.9 μg	1 dose
3-9 year olds	Q-Pan0.9	A/California/7/2009 (H1N1)v-like + AS03_B_	0.9 μg	1 dose
	Q-Pan1.9	A/California/7/2009 (H1N1)v-like + AS03_B_	1.9 μg	1 dose

*HA* haemagglutinin antigen, *AS03* an oil-in-water emulsion containing DL-α-tocopherol and squalene in an aqueous phase with the non-ionic detergent polysorbate80. AS03_A_ contains 11.86 mg DL-α-tocopherolper dose; AS03_B_ contains 5.93 mg DL-α-tocopherol per dose.

The study in children (114495, NCT01161160) was a Phase II randomised, controlled study conducted in 2 centres in the Philippines and Thailand between 25 January 2010 and 31 January 2011. Healthy children 3 to <10 years of age were randomised (13:13:10) to receive a single dose of D-Pan or Q-Pan vaccine containing one half of the recommended HA dose for children (0.9 μg HA with AS03_B_): D-Pan0.9 group and Q-Pan0.9 group), or a standard paediatric dose (1.9 μg HA with AS03_B_: Q-Pan1.9 group, Table 1).

Both of the studies were observer-blind: that is, the vaccinee and those responsible for the evaluation of any study endpoint were unaware of which vaccine was administered.

The studies were conducted according to good clinical practice and in accordance with the Somerset West 1996 version of the Declaration of Helsinki. The protocol and associated documents were reviewed and approved by local ethics committees: study in adults - in Germany: the Ethik-Kommission der Sächsische Landesärztekammer, Ethik-Kommission der Medizinischen Fakultät der Universität Würzburg, Geschäftsstelle der Ethikkommissionan der Universität Regensburg, and in France the Comité de Protection des Personnes Ile de France I. Study in children – The Royal Thai Army Medical Department Phramongkutklao Hospital in Thailand, and the Mary Chiles General Hospital in the Philippines. Written informed consent was obtained from subjects or the parents/guardians of children before study procedures.

### Study subjects

Adults were not eligible if they had clinical or confirmed influenza infection within 6 months prior to study start, if they had a history of neurological disease or Guillain-Barre syndrome, or if they had received any non-study vaccine within 30 days of enrolment. Women enrolled in the study were to agree to avoid pregnancy for 2 months after the second dose. Children were not included if they had a history of physician-confirmed infection or previous vaccination against A/California/7/2009 (H1N1)v-like virus. Other exclusion criteria included receipt of any licensed live-attenuated vaccine within 30 days before study vaccination, any licensed inactivated vaccine within 15 days of study vaccination, or planned administration of any other vaccine not foreseen by the study protocol between Day 0 and Day 21. Routine childhood vaccinations were allowed during the study, but were not to be administered on the same day as the study vaccine.

In both studies subjects were not eligible to participate if they had a diagnosis of cancer or had received treatment for cancer in the last 3 years. Subjects were not eligible if they were immunosuppressed from any cause, including chronic (>14 days) intake of immunosuppressants, if they had received blood products within 3 months of the study, or if they had any disorder of coagulation.

### Vaccines

The study vaccines were monovalent, split-virion, inactivated influenza A (H1N1) 2009 vaccines (reassortant X-179A strain derived from the A/California/7/2009 (H1N1)v virus) prepared from virus propagated in the allantoic cavity of embryonated hens’ eggs. The manufacturing processes for the antigen component of D-Pan and Q-Pan H1N1 were similar to the manufacturing processes of their corresponding licensed seasonal influenza vaccines (*Fluari*x™ and *FluLaval*™, respectively). For D-Pan H1N1, the virus was purified by centrifugation and disrupted with sodium deoxycholate. The virus was inactivated by sodium deoxycholate and formaldehyde. The split virus was further purified by ultrafiltration and sterilised by filtration. For Q-Pan H1N1, the virus was treated with ultraviolet light followed by formaldehyde inactivation. After purification by centrifugation and disruption with sodium deoxycholate, the split virus was homogenised and sterilised by filtration. All subjects received a specified volume of an antigen formulation with a concentration of HA of 15 μg/ml mixed with AS03 (Table 1).

### Immunogenicity assessment

Adults provided blood samples before and 21 days after each vaccine dose, and again 6 months and 1 year after the first dose. Blood samples were collected from children prior to vaccination, and 21 days and 6 months after vaccination. The humoral immune response to vaccination was assessed by measuring antibody inhibition of haemagglutination (HI) against the A/California/7/2009 (H1N1)v-like strainas previously described [13]. The lowest dilution tested was 1:10. The titration end-point was the highest dilution step that showed complete inhibition (100%) of haemagglutination. HI antibody titres of ≥1:40 and were considered indicative of seroprotection in adults [14,15].

### Safety and reactogenicity assessment

In each study local and age-appropriate general symptoms were solicited and their occurrence were recorded on diary cards for 7 days after each vaccine dose (day 0-6). All other adverse events in adults were recorded from study start until 63 days after the second dose. In children all other adverse events were recorded for 42 days after vaccination. Serious adverse events (SAEs) and potentially immune-mediated diseases (pIMDs) were recorded throughout the duration of the studies: for 12 months after the first dose in adults and for 6 months after vaccination in children.

### Immunogenicity objectives

#### Study in adults

The primary objective of the adult study was to demonstrate equivalence between Dresden and Quebec-manufactured A/California/7/2009 (H1N1)v-like vaccine in terms of the HI geometric mean antibody titre (GMT) ratio (D-Pan divided by Q-Pan) 21 days after dose 1. Equivalence was demonstrated if the limits of the 2-sided 95% confidence interval (CI) on the GMT ratio were within the interval [0.5:2].

Secondary objectives included assessment of the equivalence between groups in terms of HI GMTs 21 days after dose 2, and in terms of seroconversion rate 21 days after dose 1. Equivalence was demonstrated if the 2-sided 95% CI on the GMT ratio (after dose 2) was within the interval [0.5;2], and if the 2-sided 95% CI on the difference between groups in the seroconversion rate (defined below) was within the interval [-10%;10%].

#### Study in children

The co-primary objectives of the study in children were to demonstrate immunogenicity of the reduced antigen content A/California/7/2009 (H1N1)v-like vaccines (Q-Pan0.9 and D-Pan0.9 groups) in terms of Food and Drug Administration Center for Biologics Evaluation and Research (CBER) [16], and European Medicines Agency Committee for Human Medicinal Products (CHMP) [17], criteria for acceptable immunogenicity of pandemic influenza vaccines. Secondary objectives included the demonstration of equivalence between Q-Pan0.9 and D-Pan0.9 in terms of HI antibody GMTs 21 days after vaccination. Equivalence was demonstrated if the 2-sided 95% CI on the GMT ratio were within the interval [0.5;2]. Immunogenicity of the Q-Pan1.9 vaccine was also assessed.

### Statistical methods

The primary immunogenicity analysis was done on the according to protocol (ATP) immunogenicity cohorts at each blood sampling time point. At each time point, the ATP cohort included all evaluable subjects who met eligibility criteria, who complied with the protocol-defined procedures, who were not eliminated during the study and for whom data concerning immunogenicity measures were available.

The GMT ratio and the 95% CIs were calculated using a covariance (ANCOVA) model adjusted for age, pre-vaccination antibody titre and pre-vaccination history in adults, and for age and pre-vaccination antibody titre in children.

The seroconversion rate was defined as the percentage of initially seronegative vaccinees (HI titre < 1:10) with a post-vaccination titre ≥ 1:40; or the percentage of initially seropositive vaccinees (HI titre ≥1:10) with at least a 4-fold increase in the post-vaccination titre. The seroconversion factor was defined as the geometric mean of the post-vaccination titre divided by the pre-vaccination titre.

With 144 evaluable subjects in each group and assuming a standard deviation on the HI GMT of 0.65, the adult study had 95.05% power to meet the primary objective of demonstrating equivalence.

With 180 evaluable subjects and assuming a seroprotection rate of 90%, a seroconversion rate of 80% and a seroconversion factor of 20, the study in children had 94.6% power to meet the co-primary objectives of achieving CBER/CHMP criteria for D-Pan0.9 and Q-Pan0.9.

Analyses were performed using SAS® software version 9.2 (SAS Institute Inc., Cary, NC, United States) and ProcStatXact 8.1.

## Results

### Study subjects

**Figure 1:**
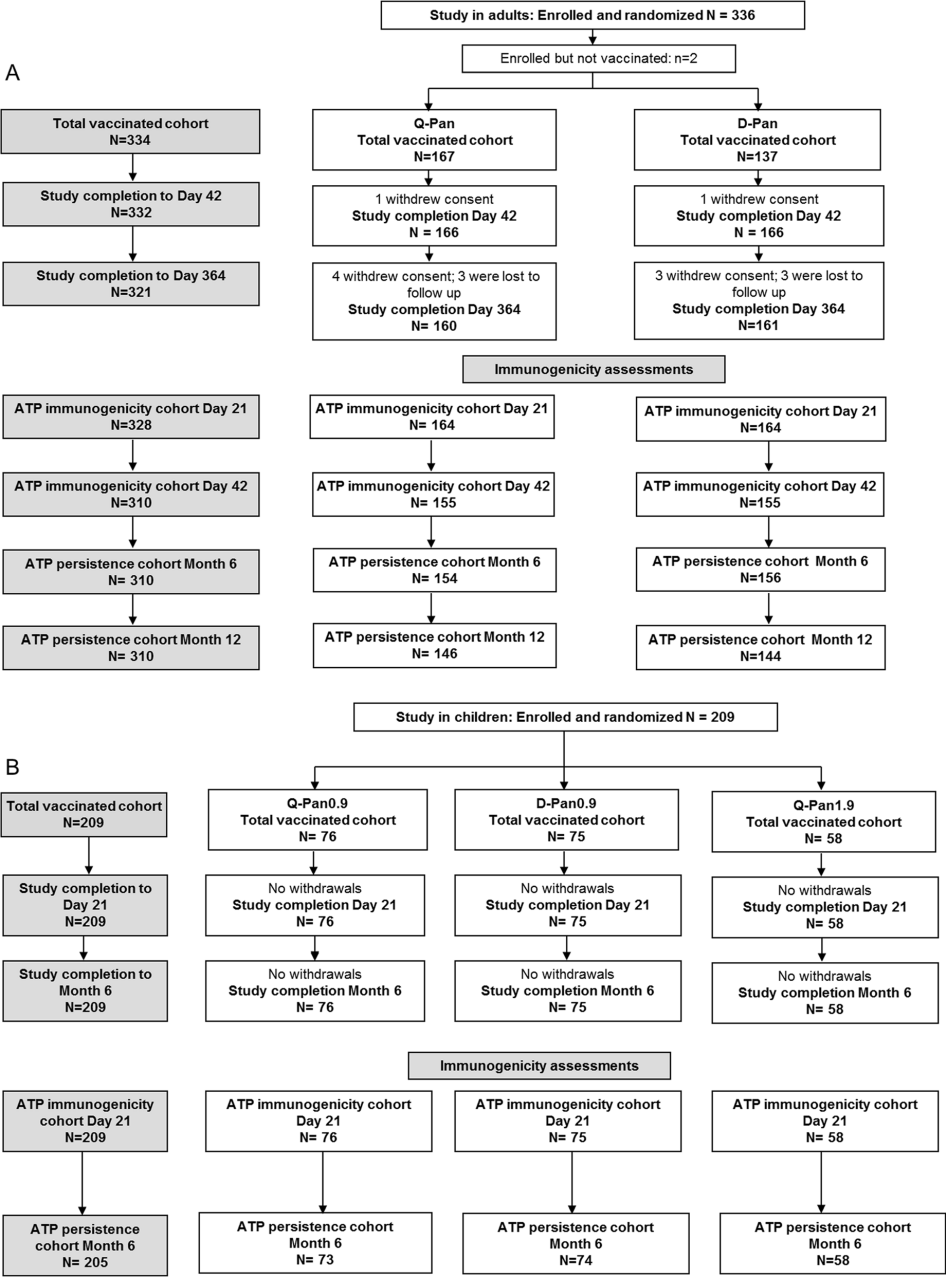
**Subject flow through the studies. (A)** Study in adults: reasons for elimination from the ATP immunogenicity and persistence cohorts were: non-compliance with vaccination/blood sampling schedule; blood sample not taken/insufficient quantity for any test; randomisation failure; received a vaccine forbidden by the protocol. **(B)** Study in children: reasons for elimination from the ATP persistence cohort were: received medication forbidden per protocol; blood sample not taken or quantity not sufficient for any test.

There were 334 subjects enrolled and vaccinated in the adult study and 209 who were enrolled and vaccinated in the study in children (Figure 1). Demographic characteristics were comparable between groups in each study (Table 2). No subject withdrew from the study due to an adverse event.

**Table 2:** Demographic characteristics: ATP immunogenicity cohorts at Day 21 in both studies

**Characteristic**	**Categories**	**Study in adults**		**Study in children**		
		**Q-Pan**	**D-Pan**	**Q-Pan0.9**	**D-Pan0.9**	**Q-Pan1.9**
**N**		164	164	76	75	58
**Age (years)**	Mean (SD)	39.7 (11.98)	40.1 (11.74)	6.0 (2.03)	6.0 (2.02)	6.0 (2.00)
	Range	18-60	19-60	3-9	3-9	3-9
**Gendern (%)**	Female	75 (45.7)	86 (52.4)	30 (39.5)	38 (50.7)	27 (46.6)
	Male	89 (54.3)	78 (47.6)	46 (60.5)	37 (49.3)	31 (53.4)
**Racen (%)**	African/African American	3 (1.8)	2 (1.2)	0	0	0
	Central/South/East & Southeast Asian	0	0	76 (100)	75 (100)	56 (100)
	Arabic/North African	3 (1.8)	0	0	0	0
	Caucasian/European	158 (96.3)	162 (98.8)	0	0	0

*N* number in the specified cohort, *n (%)* number (percentage), *SD* standard deviation. See Table 1 for details of treatment groups in each study.

### Immunogenicity in adults

The primary and secondary objectives of the adult study were met: the pre-defined criteria for equivalence of Q-Pan and D-Pan vaccines were achieved at Day 21 and at Day 42 (Table 3).

**Table 3:** Results of the inferential analysis comparing groups in the study in adults and in children (ATP immunogenicity cohorts)

**Endpoint**	**Criteria to meet the primary objectives**	**Value (95% CI)**	**Criteria met?**
**Study in adults**			
Anti-H1N1 GMTs	95% CI for ratio is within [0.5; 2] at Day 21	1.20 (0.96;1.49)	Yes
	95% CI for ratio is within [0.5; 2] at Day 42	0.9 (0.76; 1.06)	Yes
Seroconversion rate	95% CI for the difference in within [-10; +10] at Day 21	3.66 (-0.82; 8.74)	Yes
**Study in children**	(each group)		
CHMP	Seroconversion rate >40%	≥98.7% for each group	Yes
	%(≥1:40) > 70%	≥98.3% for each group	Yes
	Seroconversion factor >2.5	≥25.7 for each group	Yes
CBER	LL of the 95% CI on the seroconversion rate >40%	≥90.8 for each group	Yes
	LL of the 95% CI on the % ≥1:40 is >70%	≥ 90.8 for each group	Yes
Anti-H1N1 GMTs	95% CI for ratio (Q-Pan0.9/D-Pan0.9) is within [0.5; 2] at Day 21	0.96 (0.73; 1.26)	Yes

*95*% *CI* 95 percent confidence interval, *LL* lower limit of the 95% CI, *GMT* geometric mean antibody titre. See Table 1 for details of treatment groups in each study.

After a single vaccine dose, all subjects in both groups were seropositive for HI antibodies and at least 97.6% had titres ≥1:40 (Table 4). HI antibody GMTs increased by at least 32-fold after the first dose administered to both groups. After dose 2, 100% of subjects had titres ≥1:40 and HI antibody GMTs increased by 63-fold in each group. CHMP and CBER regulatory acceptance criteria for influenza vaccines were exceeded by both groups at Day 21 and Day 42 (Table 4).

**Table 4:** Study in adults: haemagglutinin inhibition (HI) antibodies to A/California/7/2009 (H1N1)v-like strain after vaccination (ATP cohorts for immunogenicity and persistence)

**Group**	**Time**	**N**	**Seroconversion rate**		**Seroconversion factor**		**Seroprotection rate**	
	**point**		**%**	**(95% CI)**	**Ratio**	**(95% CI)**	**%≥1:40**	**(95% CI)**
Q-Pan	Pre	164	-	-	-	-	13.4	(8.6; 19.6)
	Day 21	164	93.9	(89.1; 97.0)	32.0	(26.5; 38.6)	97.6	(93.9; 99.3)
	Day 42	155	98.7	(95.4; 99.8)	63.2	(52.6; 75.9)	100	(97.6; 100)
	Month 6	154	91.6	(86.0; 95.4)	21.7	(18.1; 25.9)	97.4	(93.5; 99.3)
	Month 12	146	72.6	(64.6; 79.7)	11.0	(9.1; 13.3)	82.9	(75.8; 88.6)
D-Pan	Pre	164	-	-	-	-	11.6	(7.1; 17.5)
	Day 21	164	97.6	(93.9; 99.3)	41.5	(34.3; 50.2)	100	(97.8; 100)
	Day 42	155	99.4	(96.5; 100)	63.0	(52.2; 76.1)	100	(97.6; 100)
	Month 6	156	92.3	(86.9; 96.0)	22.0	(18.5; 26.1)	96.8	(92.7; 99.0)
	Month 12	144	75.7	(67.9; 82.4)	11.0	(9.2; 13.2)	84.0	(77.0; 89.6)

*N* number of subjects with available results (for seroconversion rate and seroconversion factor, N - the number of subjects with pre- and post-vaccination results available), % - percentage of subjects; 95% CI - 95% confidence interval; Seroconversion: For initially seronegative subjects (i.e., HI titres <1:10), antibody titre ≥ 1:40 after vaccination. For initially seropositive subjects, post-vaccination HI titre ≥ 4 fold the pre-vaccination antibody titre. Seroconversion Factor -mean[log_10_(post-vaccination GMT/pre vaccination GMT)]; Pre = prior to vaccination, Day 21 etc- 21 days post vaccination. CBER Criteria were fulfilled if: the lower limit of the 95% CI for SCR was >40%, and the lower limit of the 95% CI for % ≥1:40 was >70%. CHMP Criteria were fulfilled if: the point estimate for SCR was > 40% and, the post-vaccination point estimate for % ≥1:40 was >70% and, the point estimate for SCF was > 2.5. See Table 1 for details of treatment groups.

**Figure 2:**
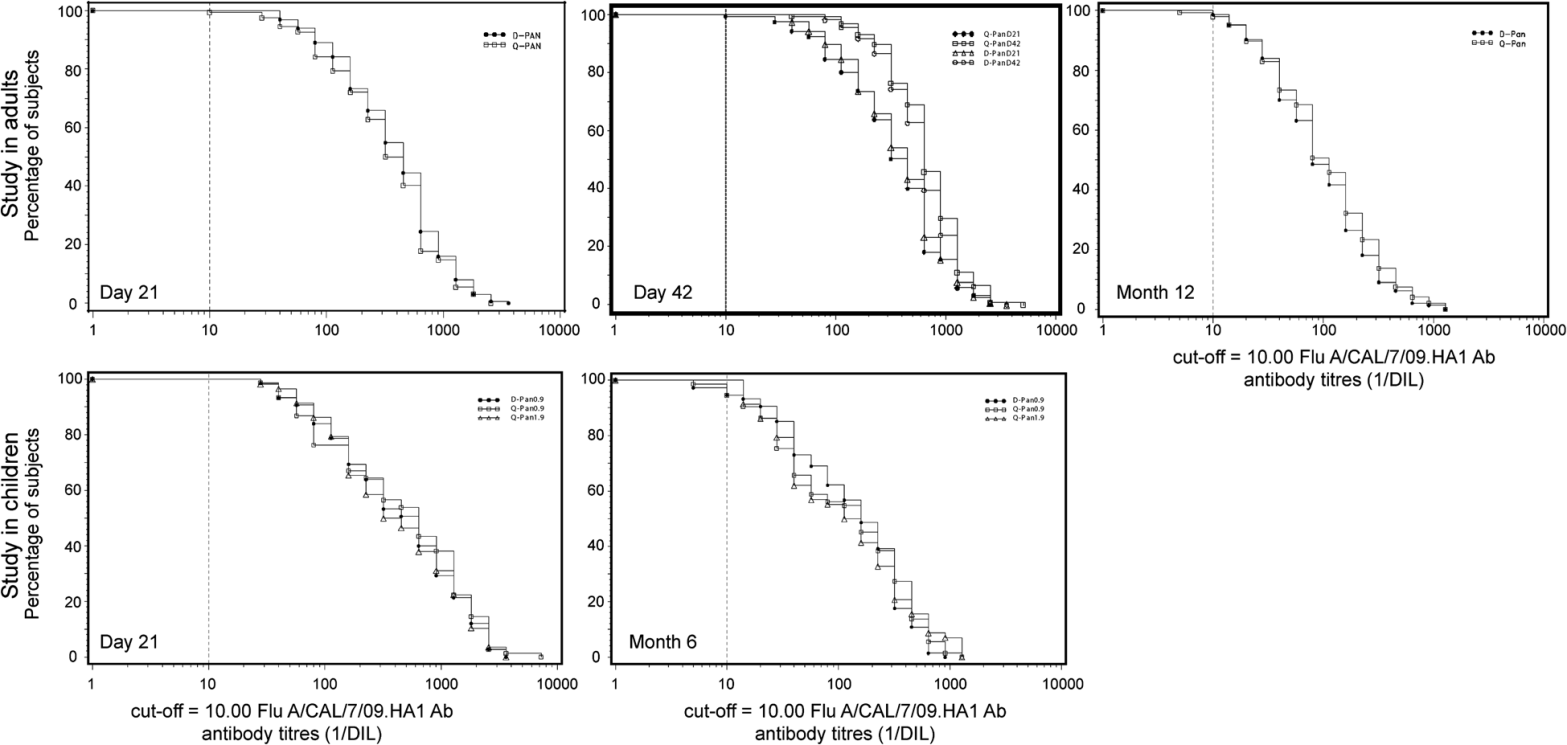
**Reverse cumulative curves of haemagglutinin inhibition (HI) antibody titres in adults and children (ATP immunogenicity cohorts for each time point).** See Table 1 for details of treatment groups in each study.

At Month 6 at least 96.8% of subjects in each group continued to have HI antibody titres ≥1:40 (Table 4). By Month 12, 82.9% of subjects in the Q-Pan group and 84.0% in the D-Pan continued to have HI antibody titres ≥1:40. GMTs reduced over time in both groups but remained higher at Month 12 than pre-vaccination levels (Figure 2).

### Immunogenicity in children

The primary and secondary objectives of the study in children were met: CHMP and CBER regulatory acceptance criteria for influenza vaccines were exceeded in both groups at Day 21 (Table 3). Equivalence between the Q-Pan0.9 and D-Pan0.9 groups was demonstrated in terms of GMTs.

At day 21 after vaccination all subjects were seropositive for HI antibodies and at least 98.3% of subjects in each group had HI antibody titres ≥1:40 (Table 5). Compared to pre-vaccination levels, GMTs at Day 21 increased by 25.7-fold in the Q-Pan0.9 group, 27.1-fold in the D-Pan0.9 group and 32.2-fold in the Q-Pan1.9 group.

**Table 5:** Study in children: haemagglutinin inhibition (HI) antibodies to A/California/7/2009 (H1N1)v-like strain after vaccination (ATP cohorts for immunogenicity and persistence)

**Group**	**Time**	**Seroconversion rate**			**Seroconversion factor**			**Seroprotection rate**		
	**point**	**N**	**%**	**(95% CI)**	**N**	**Ratio**	**(95% CI)**	**N**	**% ≥1:40**	**(95% CI)**
Q-Pan0.9	Pre	-	-	-	-	-	-	76	36.8	(26.1; 48.7)
	Day 21	76	98.7	(92.9; 100)	76	25.7	(20.7; 32.0)	76	98.7	(92.9; 100)
	Month 6	73	63.0	(50.9; 74.0)	73	6.6	(5.4; 8.2)	73	75.3	(63.9; 84.7)
D-Pan0.9	Pre	-	-	-	-	-	-	75	32.0	(21.7; 43.8)
	Day 21	75	98.7	(92.8; 100)	75	27.1	(22.4; 32.8)	75	98.7	(92.8; 100)
	Month 6	74	71.6	(59.9; 81.5)	74	8.0	(6.4; 10.1)	74	85.1	(75.0; 92.3)
Q-Pan1.9	Pre	-	-	-	-	-	-	58	31.0	(19.5; 44.5)
	Day 21	58	98.3	(90.8; 100)	58	32.2	(24.7; 42.0)	58	98.3	(90.8; 100)
	Month 6	58	69.0	(55.5; 80.5)	58	8.9	(6.8; 11.7)	58	79.3	(66.6; 88.8)

*N* number of subjects with available results (for seroconversion rate and seroconversion factor N - the number of subjects with pre- and post-vaccination results available); % - percentage of subjects; 95% CI - 95% confidence interval. Seroconversion: For initially seronegative subjects (i.e., HI titres <1:10), antibody titre≥ 1:40 after vaccination. For initially seropositive subjects, antibody titre after vaccination ≥ 4 fold the pre-vaccination antibody titre. Seroconversion Factor - Geometric Mean Ratio (mean[log_10_(post-vaccination GMT/pre vaccination GMT]). Pre - prior to vaccination, Day 21 etc- 21 days post vaccination. CBER Criteria were fulfilled if: the lower limit of the 95% CI for SCR was >40%, and the lower limit of the 95% CI for % ≥1:40 was > 70%. CHMP Criteria were fulfilled if: the point estimate for SCR was > 40% and, the post-vaccination point estimate for % ≥1:40 was > 70% and, the point estimate for SCF was >2.5. See Table 1 for details of treatment groups.

At Month 6, 75.3% of subjects in the Q-Pan0.9 group, 85.1% in the D-Pan0.9 group and 79.3% in the Q-Pan1.9 group continued to have HI antibodies ≥1:40 (Table 5). GMTs decreased over time in all groups but remained higher than pre-vaccination levels (Figure 2).

### Safety

**Figure 3:**
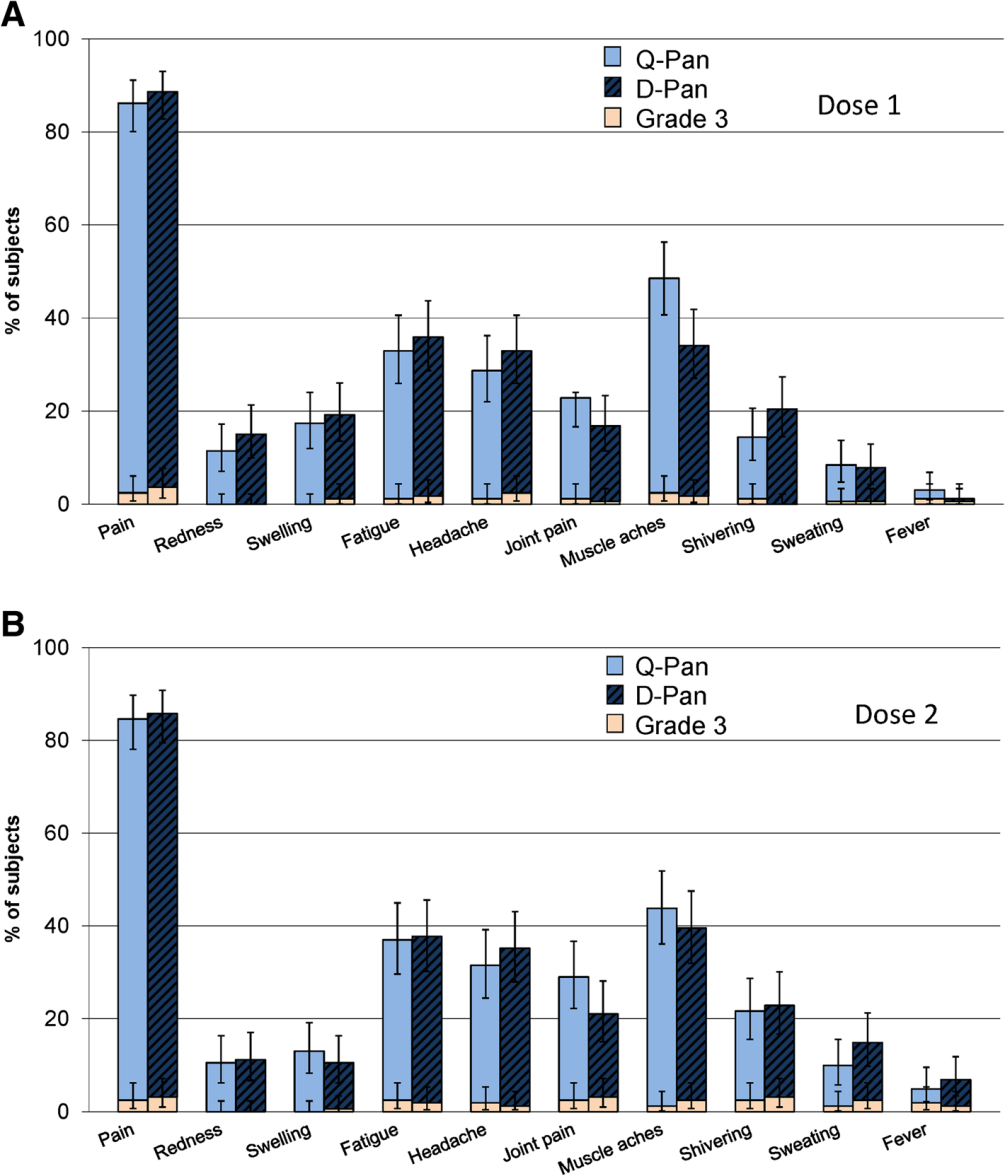
**Local and general solicited symptoms in adults.** Total vaccinated cohort within 7 days after dose 1 **(A)** and dose 2 **(B)**. Vertical lines show 95% CIs. Grade 3 was defined as: pain -significant pain at rest, prevented normal activities as assessed by inability to attend/do work or school: redness or swelling >100 mm; Fever: oral/axillary temperature ≥39.0°C; all other symptoms: Prevents normal everyday activities as assessed by inability to attend/do work or school, or requires intervention of a physician/healthcare provider. See Table 1 for details of treatment groups in each study.

**Figure 4:**
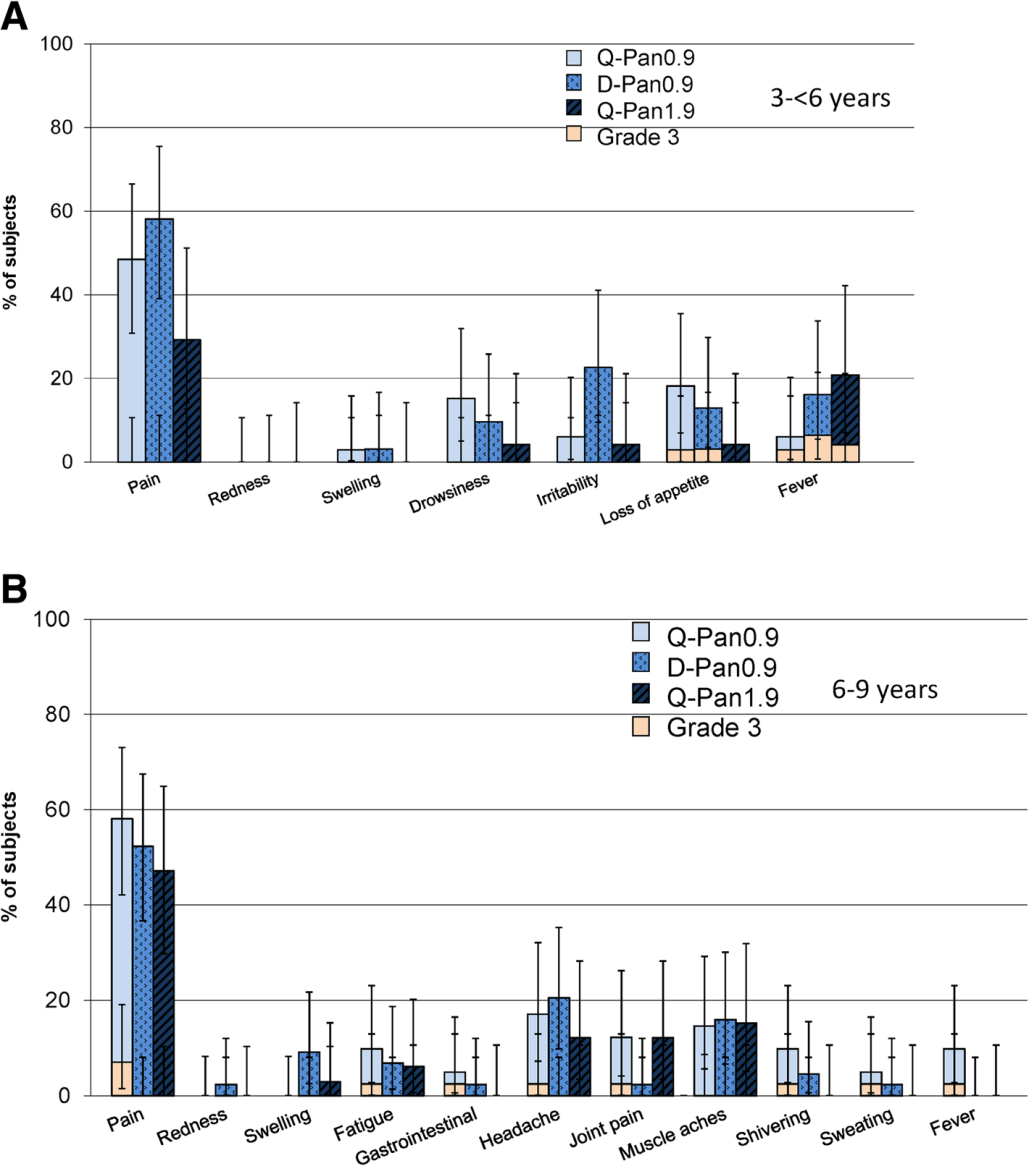
**Local and general solicited symptoms in children.** Total vaccinated cohort within 7 days after vaccination in children 3-<6 years **(A)** and in children 6-9 years **(B)**. Vertical lines show 95% CIs. Grade 3 was defined as: pain - cries when limb is moved/spontaneously painful; redness or swelling >100 mm; Fever: oral/axillary temperature ≥39.0°C; In addition, in children 3- < 6 years: Irritability - crying that cannot be comforted/prevents normal activity; Drowsiness - prevents normal activity; Loss of appetite - not eating at all. In children 6-9 years: all other symptoms - prevents normal activity. See Table 1 for details of treatment groups in each study.

The incidences of local and general solicited symptoms were generally comparable in each study across the study groups (Figures 3 and 4). Symptoms of grade 3 intensity were reported by not more than 3.6% of adults in each study group, and by not more than 6.5% of children in each age subgroup. The point estimates for each solicited local and general symptom tended to be lower in the Q-Pan1.9 group than the 2 groups who received 0.9 μg of vaccine antigen, although the 95% CIs overlapped in all cases.

Other (unsolicited) adverse events occurring until 63 days following dose 2 were reported by 80 adults in the Q-Pan group (24.2%; 95% CI 19.7, 29.2) and 86 in the D-Pan group (26.1%; 95% CI 21.5, 31.2). Of these, adverse events that were considered by the investigator to be causally related to vaccination were reported by 20 adults (12%) in each treatment group. Most were reactions at the injection site. In children, unsolicited adverse events until day 42 post-vaccination were reported by 28 subjects in the Q-Pan0.9 group (36.8%; 95% CI 26.1, 48.7), 28 subjects in the D-Pan0.9 group (37.3%; 95% CI 26.4, 49.3) and by 23 subjects in the Q-Pan1.9 group (39.7%; 95% CI 27.0, 53.4). Of these, adverse events reported by two subjects each in the Q-Pan0.9 and D-Pan0.9 groups (2.6% and 2.7%, respectively) were considered vaccine-related. No vaccine-related adverse events were reported in the Q-Pan1.9 group.

Twenty SAEs (11 in the Q-Pan group, 9 in the D-Pan group) were reported in the adult study up to Month 12. The events were diverse and classified under 13 MedDRA (Medical Dictionary for Regulatory Activities) system organ classes. None were considered to be related to vaccination. One subject in the adult study developed a pIMD: Bells palsy with onset 179 days after the second dose. The event was considered unrelated to vaccination.

Two SAEs were reported in children up to Month 6 (dengue haemorrhagic fever and influenza type B). Neither event was considered to be related to vaccination. No pIMDs were reported during the study period.

## Discussion

Achieving sufficient production to supply vaccine globally during an influenza pandemic remains an objective of the WHO and of vaccine manufacturers. In the present studies immunological equivalence was demonstrated between A/California/7/2009 (H1N1)v-like HA manufactured at two manufacturing sites when adjuvanted with AS03 in two populations (adults and children), and at two doses (standard dose in adults and low dose in children). These data allow for increased flexibility in vaccine supply in response to a pandemic, and add to reports of immunogenicity equivalence for D-Pan and Q-Pan H5N1 vaccines [12] and the use of GlaxoSmithKline’s AS03 adjuvant system with Sanofi Pasteur’sH1N1 antigen [18].

The recommended paediatric dose of A/California/7/2009 (H1N1)v-like-AS03 vaccine is half the antigen and adjuvant content of the adult dose. This study showed that reducing the HA dose further, to one-quarter of the adult dose adjuvanted with AS03_B_, resulted in robust immune response that fulfilled all CBER and CHMP criteria in children. Antibody persistence 6 months after vaccination was similar in all children, regardless of antigen dose administered. These data suggest that there may be scope to further reduce the paediatric H1N1 dose, allowing further antigen sparing (16-fold for children 3 years of age or older). These data also suggest that changing end-of-shelf-life specifications such that vaccine at expiry contains one-half the recommended antigen dose, is unlikely to impact clinical potency, but would allow for the extended use of available vaccine stocks.

The results of these studies are consistent with previously published data of A/California/7/2009 (H1N1)v-like-AS03 in adults and children [5-8,19]. Notably, a single vaccine dose was highly immunogenic in both age groups, confirming recommendations for one dose across all ages.

The reactogenicity and safety profile of A/California/7/2009 (H1N1)v-like-AS03 manufactured in Dresden and Quebec was consistent with the known reactogenicity profile of A/California/7/2009 (H1N1)v-like vaccine adjuvanted with AS03 in adults and children [3]. Reactogenicity was not observed to increase after the second dose in adults. Reports from epidemiological studies conducted in some European countries indicated a 6- to 13-fold increased risk of narcolepsy in children/adolescents vaccinated with *Pandemrix*™ (D-Pan) as compared with unvaccinated individuals [20-22]. This risk increase has not been found in adults (20 years and older). In the current studies no safety concerns were identified during extended safety follow-up for 6 months after vaccination in children and 12 months in adults, and no cases of narcolepsy or Guillain-Barré syndrome were identified [23].

The present studies provide no data on the use of D-Pan versus Q-Pan in the age group between 10 and 18 years, or in adults over 60 years of age. However, good immunogenicity of A/California/7/2009 (H1N1)v-AS03 vaccine manufactured in Quebec and Dresden has been demonstrated in other studies for both age groups [7,9,10,24-27].

## Conclusions

Two studies indicate that A/California/7/2009 (H1N1)v-like HA manufactured at two sites are immunologically equivalent when administered with AS03, allowing flexibility of supply during influenza pandemics. The studies confirm the robust immunogenicity of A/California/7/2009 (H1N1)v-like vaccine adjuvanted to AS03. The possibility to further reduce the administered antigen dose to children warrants additional investigation.

PANDEMRIX and AREPANRIX are trademarks of the GlaxoSmithKline group of companies.

## Abbreviations

ATP: According to protocol; AS03: Proprietary adjuvant system; CBER: Food and drug administration center for biologics evaluation and research; CI: Confidence interval; CHMP: European medicines agency committee for human medicinal products; D-Pan: A/H1N1/2009-AS03 vaccine manufactured in Dresden; GMT: Geometric mean antibody titre; HA: Haemagglutinin antigen; HI: Haemagglutination inhibition; pIMDs: Potentially immune-mediated diseases; Q-Pan: A/H1N1/2009-AS03 vaccine manufactured in Quebec; SAEs: Serious adverse events.

## Competing interests

AK and JJW report no competing interests. OL: OL’s institution has received research grants from GSK and she has received travel grants to present data at scientific congress from GSK. XD: XD’s institution received research grants from Pfizer and he has received travel grants from Roche. SF: SF received a consultancy fee that is unrelated to the submitted work. WJ: WJ’s institution has received consulting fees and support for travel to meetings from GSK. He has received payments for lectures including services on speaker bureaus from GSK, Sanofi-Pasteur, MSD, Baxter, ABBOTT, Roche and Mikrogen. MM: MM has received grants and honorarium from GSK and has received travel grants for presentations from GSK, Merck Sharp and Diagnostics, Novartis, Sanofi Pasteur and United Laboratories as well as payments for development of educational presentations from different organizations in the Philippines. TFS: TFS has received payments towards consultancy and as an advisory board member from GSK. He has received travel grants and payments for development of educational presentations from GSK. VW: VW’s institution has received research grants from GSK. GZ: GZ has received payments as an advisory board member from Roche, Eli-Lilly, Pfizer, BMS, CLovis Oncology and Astra Zeneca. He has received travel and accommodation grants to participate at congresses from Roche, Eli-Lilly, Astra Zeneca and BoehringerIngelheim. VB, PL, AC, AM, PG and DWV are/were employees of GlaxoSmithKline group of companies. PL, AC, AM, PG and DWV report receiving restricted shares of GlaxoSmithKline group of companies.

## Authors’ contributions

OL, XD, WJ, AK, MM, TFS, VW, JJW and GZ were the principal or co-investigators of either study NCT00979407 or study NCT01161160, results of which are disclosed here. All of these authors contributed to conducting the study at the respective sites and to obtaining the data. VB and PL were the statisticians who analysed the data from both studies. AC, AM, PG and DWV were involved in the conceptualization and designing of either of the 2 studies and critical analysis of the data. All authors critically evaluated and commented on each draft of the manuscript and all authors approved the final version.

## Pre-publication history

The pre-publication history for this paper can be accessed here:

http://www.biomedcentral.com/1471-2334/13/435/prepub
